# Targeting SUR1/Abcc8-Type Neuroendocrine K_ATP_ Channels in Pancreatic Islet Cells

**DOI:** 10.1371/journal.pone.0091525

**Published:** 2014-03-12

**Authors:** Yumiko Nakamura, Joseph Bryan

**Affiliations:** Pacific Northwest Diabetes Research Institute, Seattle, Washington, United States of America; Odense University hospital, Denmark

## Abstract

ATP-sensitive K^+^ (K_ATP_) channels play a regulatory role in hormone-secreting pancreatic islet α-, β- and δ-cells. Targeted channel deletion would assist analysis and dissection of the intraislet regulatory network. Toward this end *Abcc8*/Sur1 flox mice were generated and tested by crossing with glucagon-(GCG)-cre mice to target α-cell K_ATP_ channels selectively. Agonist resistance was used to quantify the percent of α-cells lacking channels. 41% of Sur1^loxP/loxP^;GCG-cre^+^ and ∼64% of Sur1^loxP/−^;GCG-cre^+^ α-cells lacked K_ATP_ channels, while ∼65% of α-cells expressed enhanced yellow fluorescent protein (EYFP) in ROSA-EYFP/GCG-cre matings. The results are consistent with a stochastic two-recombination event mechanism and a requirement that both floxed alleles are deleted.

## Introduction

Diabetes mellitus is a major worldwide health problem increasingly understood to be a bihormonal disease characterized by dysregulation of insulin secretion from pancreatic β-cells and glucagon secretion from α-cells [1], [2]. Failure to adequately suppress glucagon secretion from α-cells following a meal contributes to the pathogenesis of type 2 diabetes mellitus. Impaired glucagon counter-regulation and the fear of hypoglycemia is a major deterrent to maintaining tight glucose control in type 1 diabetes mellitus. The control of insulin and glucagon release in response to varying blood sugar is complex, hierarchical and redundant. Regulation involves inputs from the central nervous system and local control via a network of interactions between islet cells, including α-, β- and δ-cells [1], [3], [4]. Our understanding of the local control network is inadequate at the cellular and molecular levels and there are few tools or mouse models available to dissect network interactions. K_ATP_ channels can act as metabolic sensors in α-, β- and δ-cells. Closure of K_ATP_ channels in β- and δ-cells, secondary to increased glucose metabolism, potentiates insulin and somatostatin release, respectively. The role of these channels in α-cells is more controversial. Opening of channels during hypoglycemia has been proposed to be necessary for glucagon secretion ([5] reviewed in [6]). Alternatively the intra-islet insulin hypothesis proposes that the paracrine actions of β-cell secretion suppress glucagon release [7], [8], potentially via a K_ATP_-dependent mechanism ([9], reviewed in [10]). Glucagon is reported to have small local stimulatory effects on release of somatostatin (reviewed in [11]). Somatostatin and somatostatin analogs are used clinically to inhibit multiple functions including insulin release. Recent studies show local somatostatin release attenuates the secretion of both insulin and glucagon (for example [12], [13], [14], [15]).

The availability of mouse models selectively targeting islet cell K_ATP_ channels should aid the dissection of network interactions by uncoupling hormone release from glucose metabolism. Thus SUR1 flox mice, in which exon 2 of the *Abcc8*/Sur1 (ATP binding cassette C8/Sulfonylurea receptor type 1) gene is flanked by *loxP* sites, were generated with the intention of targeting K_ATP_ channels in select islet cell types. Exon 2 was targeted to complement Sur1^−/−^ mice in which exon 2 deletion globally eliminates SUR1 neuroendocrine type K_ATP_ channels [16]. In comparison with the severe hypoglycemia characteristic of patients with congenital hyperinsulinism (CHI) secondary to loss of K_ATP_ channel function (reviewed in [17]), Sur1^−/−^ mice, with the equivalent channel deficit, show near normal glucose homeostasis unless stressed [16], [18], [19], [20]. The mechanism(s) of compensation in the mouse model are unclear, but may reflect differences in human versus mouse islet architecture and thus differences in network feedback loops in addition to differences in the glucose-dependent amplification pathway [21].

The successful generation of these mouse models requires that sufficient, ideally all, of the targeted islet cells lack SUR1. Unlike inactivation of a gene where haploinsufficiency produces loss of function, studies of Sur1^−/−^ and Sur1^+/−^ islet cells show that deletion of *both* exon 2 alleles is required to eliminate K_ATP_ channels. Previous studies showed the number of channels in Sur1^+/−^ β-cells was indistinguishable from wildtype (WT), while Sur1^−/−^ β-cells showed a complete loss [16]. Similarly, CHI is a recessive genetic disorder. Therefore we tested the ability of cre-recombinase to produce K_ATP_ channel deficient α-cells in Sur1^loxP/-^ and Sur1^loxP/loxP^ animals in which one or two recombination events are needed to delete channel function, respectively.

In animal models, the frequency of single recombination events is often determined by crossing cre-recombinase into a cre-reporter mouse strain, for example ROSA26-stop-lacZ [22] or ROSA26-stop-EYFP [23], then assessing what fraction of a specific cell type expresses the reporter. Reported frequencies are often >0.8 for a single event which, assuming a random process, would give a frequency of >0.64 of targeted islet cells lacking K_ATP_ channels. To test this idea Sur1^loxP/loxP^ and Sur1^loxP/-^ animals GCG-cre mice expressing cre-recombinase under control of the glucagon promoter [24] were used to generate Sur1^loxP/loxP^;GCG-cre^+^ and Sur1^loxP/-^;GCG-cre^+^ mice. The frequency of channel-deficient α-cells was compared with the single event frequency for expression of EYFP in α-cells from ROSA-stop-EYFP GCG-cre crosses. EYFP was expressed in ∼65% of α-cells, while ∼41% of Sur1^loxP/loxP^;GCG-cre^+^ α-cells showed complete loss of K_ATP_ channels versus 64% in Sur1^loxP/-^;GCG-cre^+^ α-cells. The results are consistent with a stochastic two-hit mechanism and provide two animal models with varying levels of K_ATP_ channel deficient α-cells.

## Materials and Methods

All of the animal studies were approved by the Institutional Animal Care and Use Committee of the Pacific Northwest Diabetes Research Institute. The Pacific Northwest Diabetes Research Institute has an approved Animal Welfare Assurance on file with the Office for Laboratory Animal Welfare (A3357-01). Animals were maintained with a 12-h light-dark cycle at constant temperature (22±2°C) and were given free access to food and water.

### 

#### Generation of Sur1^loxP/loxP^ mice

**Figure 1:**
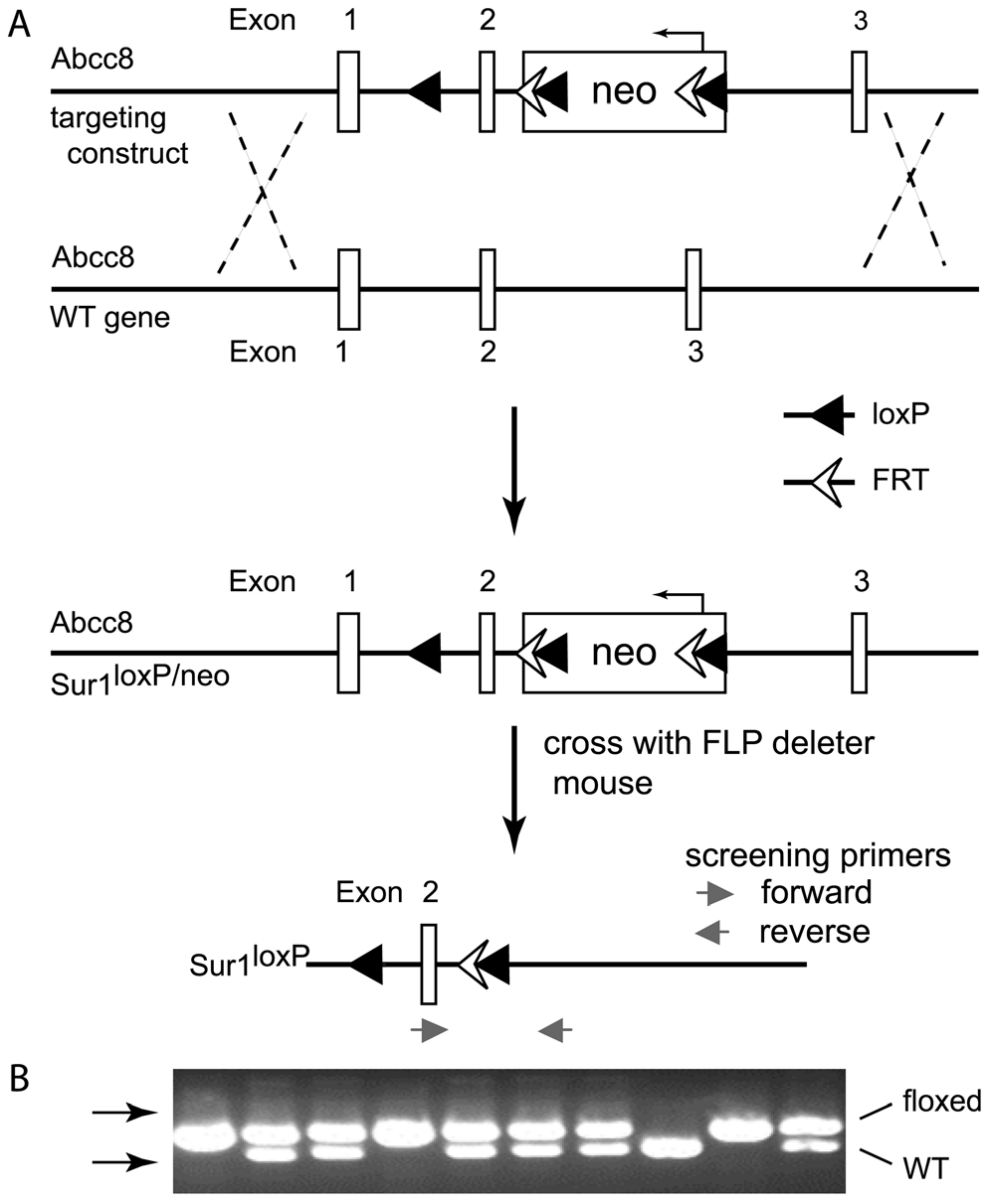
Conditional targeting strategy to create Sur1 flox mice. (A) Illustration of the targeting construct and possible recombination event to produce founder mice carrying the neomycin resistance cassette. (B) Examples of PCR products from a total of 10 mice are shown; WT (lane 8), homozygous Sur1^loxP/loxP^ (lanes 1,4 and 9) and heterozygous Sur1^loxP/+^ (lanes 2,3,5,6,7 and 10). The arrows show the position of 500 and 1000 base-pair markers.

A targeting vector (Figure 1A) was constructed using a 10.63 kb region subcloned from a C57BL/6 BAC clone (RPCI23: 301A13). The construct was designed with a long homology arm extending approximately 7.1 kb 5′ of exon 2 including exon 1 and a short homology arm extending approximately 2.59 kb 3′ of exon 2. A single *loxP* site was inserted 5′ of exon 2 and a *loxP/FRT* flanked Neo cassette was inserted on the 3′ side of exon 2. The targeted region is 928 bp including exon 2. The targeting vector was confirmed by restriction digests and by sequencing the regions of insertion. The linearized targeting vector was assembled and transfected into C57BL/6N x 129SvEv hybrid embryonic stem cells by inGenious Targeting Laboratory, Inc (Stony Brook, New York). G418, an aminoglycoside antibiotic, was used to select cells carrying the Neomycin resistance cassette. Cells were selected and correctly targeted recombinant ES cells were identified by PCR analysis. Retention of the upstream *loxP* site was confirmed by PCR analysis and by sequencing. Sur1^loxP-neo^ mice were crossed with an *FLP* deleter mouse strain (B6.Cg-Tg(ACTFLPe)9205Dym/J; Jackson Laboratories, Inc.) to eliminate the neo cassette. The possible recombinants were distinguished by PCR analysis to identify animals with the Sur1^loxP^ allele (Figure 1B). The floxed exon 2 allele is distinguished from the wild type allele using forward (5′-TGA GAT CGC TGA GGG TAT CC-3′) and reverse (5′-GGG CTG TGC ACT GTG AAT AC-3′) primers (Figure 1C). The amplified fragments are 728 bp for the floxed-allele and 551 bp for the wild type allele.

#### Generation of Sur1^loxP/loxP^;GCG-cre^+^ mice

Sur1^loxP/loxP^; *GCG*-cre^+^ animals were generated by multiple crosses of Sur1^loxP^ and GCG-cre [24] mice. The GCG-cre animals were kindly provided by Dr. Rohit Kulkarni (Joslin Diabetes Center, Boston, MA). The *loxP* exon 2 allele was identified by PCR analysis using the forward and reverse primers given above. The GCG-cre allele was identified by PCR analysis using forward (5′-ATG CTT CTG TCC GTT TGC CG-3′) and reverse (5′-CCT GGC AAT TTC GGC TAT AC3-3′) primers.

#### Generation of Sur1^loxP/-^;GCG-cre^+^ mice

Crossing Sur1^loxP/loxP^; *GCG*-cre^+^ and Sur1^−/−^ [16] generated Sur1^loxP/-^;*GCG*-cre^+^ animals. The knockout allele was identified using forward (5′-AGG TTG TTG GTG GAG GTC AG-3′) and reverse (5′-GCT ACT TCC ATT TGT CAC G-3′) primers.

#### Generation of GCG-cre-ROSA26-stop-EYFP mice

GCG-cre-ROSA26-stop-EYFP animals were generated by crossing GCG-cre and ROSA26-stop-EYFP (B6.129X_1_-Gt(ROSA)26Sor^tm1(EYFP)Cos^/J; Jackson Laboratories, Inc.) animals. The Gt(ROSA)26Sor^tm1(EYFP)Cos^ allele was identified using forward (5′-AAA GTC GCT CTG AGT TGT TAT -3′) and reverse (5′-AAG ACC GCG AAG AGT TTG TC-3′) primers.

#### Islet isolation

On the day of pancreas removal, animals were anesthetized with a ketamine (600 mg/Kg)-xylazine (50 mg/Kg) mixture and then killed by removing blood from the heart. Pancreata were cannulated for infusion of collagenase and then removed from the animal for processing as described [9] using 1 mg/ml collagenase. Islets were dissociated by mechanical dispersion in a Ca^2+^-free medium with 0.1 mM EGTA. The mix of isolated islet cells, primarily α-, β- and δ-cells, and small clusters were plated on glass cover slips and cultured overnight in RPMI medium 1640 containing 10% FBS, 11.1 mM glucose, and 100 units/ml of penicillin, 100 μg/ml of streptomycin, and 0.25 μg/ml of amphotericin B (Gibco/Life technologies, Inc).

#### Calcium Imaging

The cytoplasmic free Ca^2+^ ([Ca^2+^]_c_) concentration was measured by dual excitation-emission spectrofluorimetry using fura-2 [25] (Molecular Probes, Inc., Eugene, OR). Cells were loaded with 0.2 μM fura-2/AM for 30 min and perifused in Krebs-Ringer bicarbonate HEPES buffer (KRB-HEPES) containing (mM) 129 NaCl, 4.7 KCl, 1.2 KH_2_PO_4_, 1.2 MgCl_2_, 2 CaCl_2_, 5 NaHCO_3_, 10 HEPES (equilibrated pH 7.4) supplemented with 0.1% BSA. Measurements were carried out using a Leica DM6000B microscope. Excitation was at 340 and 380 nm; emission was recorded at 510 nm at intervals of 3 seconds. Relative [Ca^2+^]_c_ is defined as the 340/380 ratio.

#### Identification of α-cells

α- and β-cells can be discriminated functionally by their response to a 5 minute pulse of epinephrine (5 μM) in 2.8 mM glucose; α-cells showed a robust increase in [Ca^2+^]_c_, while β-cells exhibited the reverse response. This method was motivated in part by understanding of the glucagon counter-regulatory response to hypoglycemia where epinephrine hyperpolarizes β-cells and δ-cells [26] thus inhibiting their hormone release, while stimulating α-cells to secrete glucagon [27].

#### Assay for K_ATP_ channel deletion

The absence of channels was determined by assessing the response of isolated α-cells to the K_ATP_ channel agonist, diazoxide. Fura2-loaded α-cells, in KRB-HEPES with 2.8 mM glucose, identified functionally by their response to epinephrine, were perifused with 1 mM arginine ± 100 μM diazoxide. In WT α-cells arginine elevates [Ca^2+^]_c_ and will stimulate glucagon release; opening K_ATP_ channels with diazoxide completely suppresses the arginine-induced [Ca^2+^]_c_ increase. In α-cells lacking K_ATP_ channels, e.g., isolated from Sur1^−/−^ mice, the agonist has no effect. Diazoxide half-maximally stimulates SUR1/Kir6.2 K_ATP_ channels at ∼60 μM [28] therefore concentrations (10, 30 and 100 μM) bracketing this value were used.

#### Blood glucose measurements

Glucose was measured on blood from a tail vein using a Freestyle glucometer (Abbott Laboratories. Abbott Park, Illinois, U.S.A). Measurements were made in the morning on fed animals, i.e., animals given free access to food and water.

## Results

### Insertion of loxP sites does not affect the general phenotype of Sur1^loxP/loxP^ animals

The Sur1^loxP/+^ and Sur1^loxP/loxP^ animals are viable, fertile, appear phenotypically normal and have a normal lifespan. Table I compares the body weight and fed blood glucose values of WT versus Sur1^loxP/+^ and Sur1^loxP/loxP^ animals at 4 weeks of age. The results indicate insertion of *loxP* sites around exon 2 of the *Abcc8*/Sur1 gene does not have deleterious effects on development or glucose homeostasis.

**Table I:** Mouse body weight and blood glucose values.

Strain	BW (gram)	BG (mg/dl)
**Males**		
WT	18.6±0.8	135.7±10.7
Sur1^loxP/+^	18.7±0.4	140.7±5.2
Sur1^loxP/loxP^	18.0±0.7	135±4.5
**Females**		
WT	15.9±0.2	119.2±5.7
Sur1^loxP/+^	15.9±0.3	119.9±4.8
Sur1^loxP/loxP^	16.0±0.5	120.6±6.4

Mean ± SEM, n = 6∼10.

### Ca^2+^-imaging assay for presence of K_ATP_ channels in islet cells

**Figure 2:**
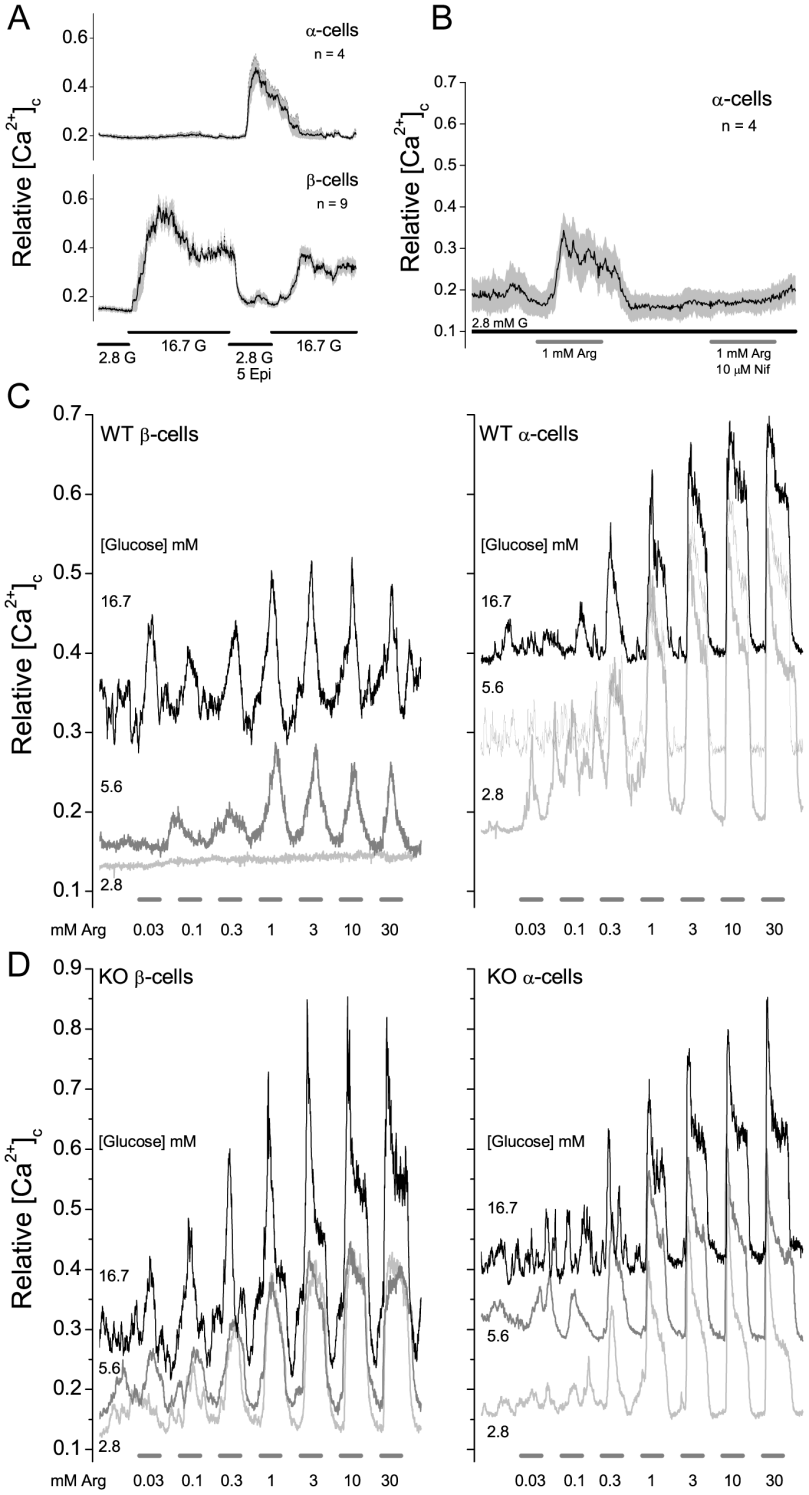
Stimulation of isolated pancreatic islet cells by arginine. (A) α- and β-cells were distinguished by their response to an epinephrine pulse in 2.8 mM glucose. (B) Arginine stimulation was blocked by nifedipine (10 μM). The solid traces and shaded areas are the means ± SEM, respectively, for the indicated number of cells. These experiments were repeated four times with similar results using different islet preparations derived from 1 or 2 mice. (C and D) Responses of WT and Sur1^−/−^ α- and β-cells to increasing concentrations of arginine at three concentrations of glucose. Each trace is an average of Ca^2+^ values from 4–10 cells; the experiments were repeated 4 times with similar results using different islet preparations. Islet preparations were from 1 or 2 mice. The pulse lengths are 5 minutes. The 5.6 and 16.7 mM glucose α-cell traces are offset 0.1 and 0.2 units respectively, for clarity.

To discriminate whether K_ATP_ channels are present, a rapid Ca^2+^-imaging assay was developed based on the arginine stimulation test used to assess the secretory capacities of α- and β-cells in patients [29], [30]. Changes in [Ca^2+^]_c_ were assessed in isolated islet cells in response to a pulse of arginine at three glucose concentrations. The effect of K_ATP_ channel loss was assessed by comparing the responses of isolated WT and Sur1^−/−^ islet cells. As indicated in Materials and Methods islet cell types were discriminated by their response to a 5 minute pulse of epinephrine (5 μM) in 2.8 mM glucose; α-cells show a robust increase in [Ca^2+^]_c_, while β-cells exhibit the reverse response (Figure 2A).

Figures 2C and D compare the responses of WT (2C) and Sur1^−/−^ (2D) α- and β-cells. Arginine stimulates a rise in [Ca^2+^]_c_ in α-cells irrespective of whether K_ATP_ channels are present (compare right panels in Figures 2C vs D). The initial values for α-cells are quite similar, therefore the traces are offset by 0.1 and 0.2 units for 5.6 and 16.7 mM glucose, respectively, to minimize overlap. The responses to 0.03 and 0.1 mM arginine are quite variable, while pulses of 0.3 mM or greater consistently increase [Ca^2+^]_c_.

In WT β-cells, under hypoglycemic conditions (2.8 mM glucose), the openings of K_ATP_ channels are sufficient to inhibit the depolarizing effect of arginine (to 30 mM; Figure 2C). When K_ATP_ channels in WT β-cells are closed by increasing the glucose concentration arginine readily increases [Ca^2+^]_c_ (Figure 2C). Similarly, Sur1^−/−^ β-cells lacking K_ATP_ channels are readily depolarized by arginine even in 2.8 mM glucose (Figure 2D). As expected, the initial β-cell [Ca^2+^]_c_ values are elevated by increasing concentrations of glucose in both WT and Sur1^−/−^ β-cells [21], [31], [32].

**Figure 3:**
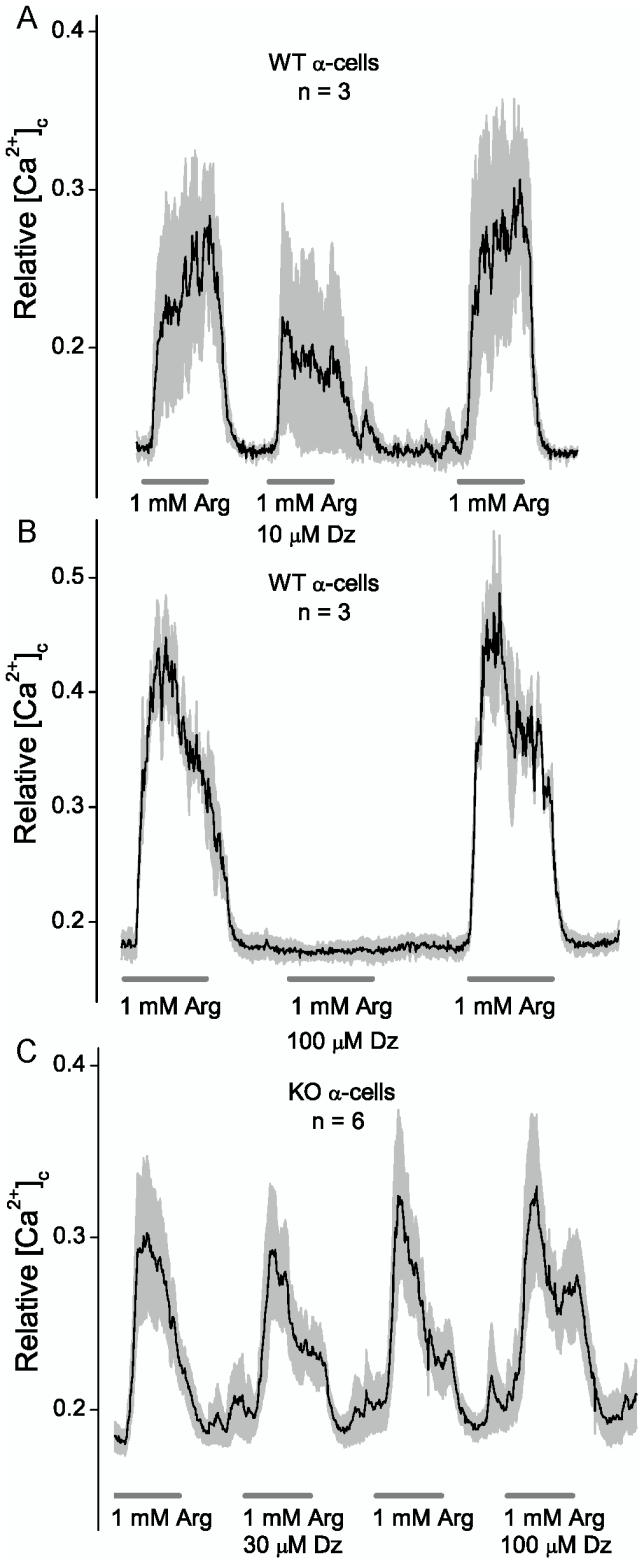
Effect of diazoxide on arginine stimulation of α-cells in WT versus Sur1^−/−^ α-cells. The cells were in 2.8± SEM, respectively, for the indicated number of cells. The experiments were repeated four times with similar results using different islet cell preparations obtained from 1 or 2 mice. The pulse lengths are 5 minutes.

We assume that arginine induces depolarization of α-cells and activates voltage-gated L-type Ca^2+^-channels since the rise in [Ca^2+^]_c_ is blocked by the L-type channel antagonist, nifedipine (10 μM; Figure 2B). Tolbutamide (200 μM) had no significant effect on arginine activation of α-cells in 2.8 mM glucose (data not shown). The results imply that under these conditions, K_ATP_ channels do not make a significant contribution to determining the membrane potential of isolated α-cells, in contrast to their role in β-cells. Therefore opening α-cell K_ATP_ channels with a channel agonist like diazoxide should reduce the stimulatory effect of arginine. Figure 3 shows that diazoxide blocks the stimulatory action of arginine in WT (Figure 3A, B), but not Sur1^−/−^ α-cells (Figure 3C). The results show that when K_ATP_ channels are present opening them with diazoxide blocks the stimulatory action of arginine. Thus the resistance to diazoxide provides an assay to identify islet cells that lack K_ATP_ channels.

### Analysis of K_ATP_ channels in Sur1^loxP/loxP^;GCG-cre^+^ and Sur1^loxP/-^;GCG-cre^+^ islet cells

**Figure 4:**
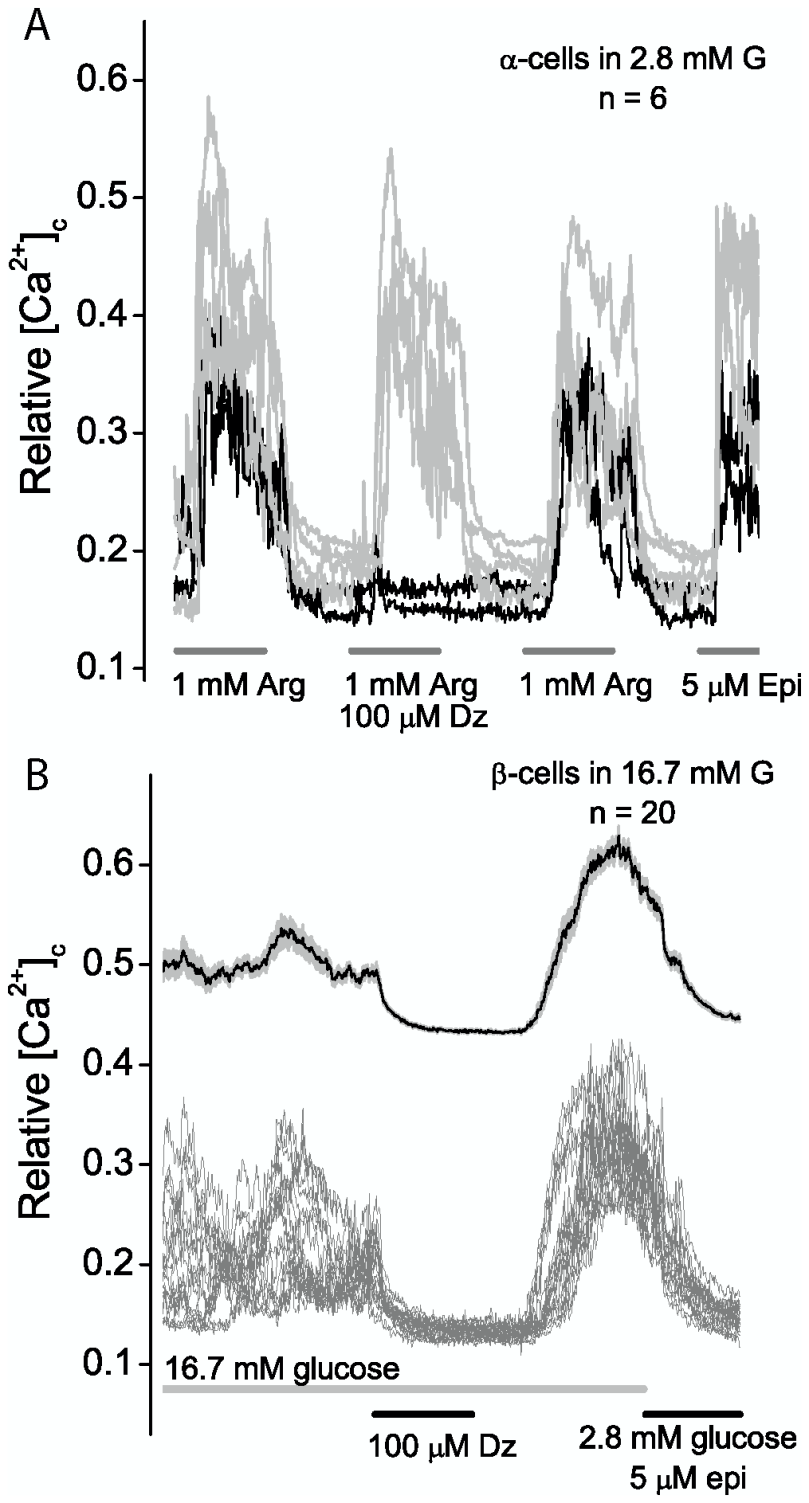
Analysis of Sur1^loxP/loxP^;GCG-cre^+^ islet cells. (A) α-Cells in 2.8 mM glucose were pulsed for five minutes with arginine (1 mM) or arginine plus diazoxide (100 μM) as indicated. In this experiment diazoxide had no effect in four cells (gray traces), while in two cells (black traces) the channel agonist blocked stimulation by arginine. All six cells were stimulated by epinephrine (5 μM). (B) In a separate experiment β-cells were stimulated with 16.7 mM glucose and pulsed for five minutes with diazoxide (100 μM). The individual traces show [Ca^2+^]_c_ was reduced to baseline values in all of the β-cells. The upper trace, offset by 0.3 units, shows the mean ± SEM values (n = 20).

Figure 4A shows an analysis of islet cells isolated from a Sur1^loxP/loxP^;GCG-cre^+^ mouse. Islet cells in 2.8 mM glucose were stimulated with arginine (1 mM) in the presence or absence of diazoxide (100 μM). The individual traces for six epinephrine-stimulated α-cells are shown. Two types of α-cells were found, those resistant and those sensitive to diazoxide (gray and black traces respectively). In this example, 4 of 6 α-cells are resistant to diazoxide, i.e., the fraction of diazoxide resistant cells was 0.67. The assumption is that both exon 2 alleles have been deleted in the diazoxide resistant cells, whereas the diazoxide sensitive cells could have one or both alleles intact. In a separate experiment epinephrine suppressed non α-cells, primarily β-cells, which were stimulated with high glucose (16.7 mM). Figure 4B shows that in 20 of 20 cells a pulse of diazoxide (100 μM) reduced [Ca^2+^]_c_. The mean ± SEM is shown in the upper trace, which is offset for clarity. The result shows that cre-recombinase is expressed selectively in α-cells.

Table II compares the fraction of diazoxide resistant α-cells in islets from Sur1^loxP/loxP^;GCG-cre^+^ and Sur1^loxP/-^;GCG-cre^+^ mice with the fraction of EYFP positive α-cells in ROSA-EYFP;GCG-cre^+^ mice (all 12–15 weeks of age). The results are consistent with the need to delete both floxed exon 2 alleles in an α-cell for complete loss of K_ATP_ channel activity and a frequency of recombination of ∼0.64 for cre-recombinase driven by the glucagon promoter. There is a small age dependence of cre-recombinase activity in this model. Sur1^loxP/-^;GCG-cre^+^ mice had a frequency of recombination = 0.68 (42/62) at 24weeks of age vs 0.64 at 12–15 weeks.

**Table II:** Frequency of recombination in Cre mouse strains.

Fraction of α-cells lacking K_ATP_ channels		Fraction of EYFP positive α-cells
Sur1^loxP/loxP^;GCG-cre^+^	Sur1^loxP/-^;GCG-cre^+^	ROSA-EYFP;GCG-cre^+^
# Dz resistant / epi positive	# Dz resistant / epi positive	# EYFP positive / epi positive
35/86∼ 0.41	45/70∼ 0.64	67/103 ∼ 0.65

epi  =  epinephrine; male mice 12-15 weeks old.

## Discussion

The objective was to develop an assay to assess the presence or absence of SUR1/Kir6.2 neuroendocrine-type K_ATP_ channels in single islet cells and use this assay to determine the efficiency of channel deletion when *Abcc8*/Sur1 flox mice are crossed with an animal expressing cre-recombinase driven by a promoter selective for pancreatic islet α-cells. In Sur1 flox mice exon 2 of the *Abcc8*/Sur1 gene is flanked by loxP sites. Deletion of both copies of exon 2 in Sur1 global knockout mice, Sur1^−/−^, eliminates their neuroendocrine-type K_ATP_ channels, while islet cells isolated from heterozygous animals have channel densities indistinguishable from WT [16]. Therefore assessing the efficiency of K_ATP_ channel deletion requires looking at individual cells. An IV bolus of arginine is used clinically to assess the secretory capacities of both α- and β-cells [29], [30], thus the effect of pulses of arginine on isolated islet cells was determined using standard Ca^2+^-imaging techniques. α- and β-cells were identified by their response to epinephrine. Under hypoglycemic conditions, 2.8 mM glucose, arginine (≤30 mM) failed to stimulate a rise in [Ca^2+^]_c_ in WT β-cells implying that under these conditions open K_ATP_ channels are sufficient to prevent arginine-induced membrane depolarization and activation of voltage-dependent Ca^2+^ channels. Closing channels, either by increasing the glucose concentration or using Sur1^−/−^ β-cells, produced a concentration-dependent response to added arginine. Arginine induced a rise in α-cell [Ca^2+^]_c_ at the three concentrations of glucose tested, thus the application of 1 mM arginine in 2.8 mM glucose was used to stimulate α-cells selectively. Tolbutamide did not affect the α-cell Ca^2+^ response to arginine significantly implying K_ATP_ channels are mainly closed in these cells even in 2.8 mM glucose. This is consistent with the observation that ATP levels as determined by NAD(P)H fluorescence are nearly unchanged in mouse α-cells at even lower glucose concentrations (0.5 mM glucose; [33]). Addition of the K_ATP_ channel agonist, diazoxide (100 μM), blocked the depolarizing action of arginine completely in WT cells showing that diazoxide resistance is a viable means to identify islet cells lacking K_ATP_ channels.

The GCG-cre mice have been used in several studies including lineage tracing studies [24], to reduce the number of insulin receptors in α-cells [34], to generate animals with fluorescent α-cells by crossing with ROSA reporter mice [35], [36], [37] and to reduce UCP2 in α-cells [38]. The glucagon promoter sequence in the GCG-cre mouse is an ∼890 basepair *SacI* fragment that ends approximately 75 basepairs upstream of the start site in glucagon [24]. The frequency of single recombination events using the GCG-cre mouse has been estimated by crossing with ROSA-lacZ or ROSA-EYFP mice. A single recombination event is required to delete the STOP codon from a loxP-STOP-loxP cassette and allow expression of the marker in these animals. The frequency estimates range from ∼0.85 [34], 0.76 [36] to 0.72±0.1 [38] determined as marker positive cells versus glucagon positive cells identified immunochemically. Using a functional assay, simulation by epinephrine, to identify α-cells we determined a value of 0.65. This was approximately equivalent to the frequency of α-cells lacking K_ATP_ channels in Sur1^loxP/-^;GCG-cre^+^ islets where excision of a single exon 2 allele should result in channel loss. The frequency, ∼0.41, of α-cells without K_ATP_ channels in Sur1^loxP/loxP^;GCG-cre^+^ islets is consistent with a need for two recombination events, i.e., (0.65×0.65∼0.42).

Diazoxide resistance can also be used to assess loss of channels in other islet cells. As shown in Figure 4B, we have not observed loss of channels in β-cells in crosses of SUR1 flox and GCG-cre animals.

The single recombination event frequency (0.64) and the observation that Abcc8 is haplosufficient, i.e., both exon 2 alleles need to be eliminated, limits the percentage of Sur1^loxP/loxP^;GCG-cre^+^ α-cells lacking K_ATP_ channels to about 41%. This is improved about two-fold in Sur1^loxP/-^;GCG-cre^+^ α-cells with a single exon 2 allele. It is not clear what limits the frequency of recombination in these animals. Araki *et al* [39] have reported a positive correlation between the level of cre-recombinase expression and frequency of recombination in a transient expression system. We have attempted, unsuccessfully, to detect cre-recombinase expression using a sensitive double immunofluorescence assay [40]. Thus a low level of enzyme expression may be a factor. It is worth noting that while excision by cre-recombinase is usually thought to be irreversible, it is an enzymatic reaction and cre technology is used in transgenesis experiments to insert selective markers at engineered loxP sites in genomic DNA (reviewed in [41], [42]). Studies are in progress to determine if higher level expression of cre-recombinase will increase the frequency of recombination and thus the percent of K_ATP_ channel deficient islet cells.

This project validates the functionality of Sur1 flox mice and provides two animal models to analyze the role of K_ATP_ channels in α-cell function *in vivo*. Based on *in vitro* studies using isolated islets opening K_ATP_ channels have been argued either to be necessary for glucagon secretion during hypoglycemia or to be stimulated and suppress glucagon release during hyperglycemia. Comparison of glucose homeostasis in these two models versus WT mice should aid in discriminating between these two hypotheses.
